# TFF1 Promotes EMT-Like Changes through an Auto-Induction Mechanism

**DOI:** 10.3390/ijms19072018

**Published:** 2018-07-11

**Authors:** Elena Romano, Megi Vllahu, Valentina Bizzarro, Raffaella Belvedere, Roberta Esposito, Antonello Petrella, Alessandra Tosco

**Affiliations:** 1 Department of Pharmacy, University of Salerno, 84084 Fisciano (SA), Italy; elena__romano@libero.it (E.R.); mvllahu@unisa.it (M.V.); vbizzarro@unisa.it (V.B.); rbelvedere@unisa.it (R.B.); roesposito@unisa.it (R.E.); 2 PhD Program in Drug Discovery and Development, University of Salerno, 84084 Fisciano (SA), Italy

**Keywords:** TFF1, gene regulation, invasion, epithelial to mesenchymal transition, hypoxia

## Abstract

Trefoil factor 1 (TFF1) is a small secreted protein expressed in the gastrointestinal tract where, together with the other two members of its family, it plays an essential role in mucosal protection and repair against injury. The molecular mechanisms involved in the protective function of all three TFF proteins are not fully elucidated. In this paper, we investigated the role of TFF1 in epithelial to mesenchymal transition (EMT) events. The effects of TFF1 on cellular models in normoxia and/or hypoxia were evaluated by western blot, immunofluorescence, qRT-PCR and trans-well invasion assays. Luciferase reporter assays were used to assess the existence of an auto-regulatory mechanism of TFF1. The methylation status of TFF1 promoter was measured by high-resolution melting (HRM) analysis. We demonstrate a TFF1 auto-induction mechanism with the identification of a specific responsive element located between −583 and −212 bp of its promoter. Our results suggest that TFF1 can regulate its own expression in normoxic, as well as in hypoxic, conditions acting synergistically with the hypoxia-inducible factor 1 (HIF-1α) pathway. Functionally, this auto-induction mechanism seems to promote cell invasion and EMT-like modifications in vitro. Additionally, exogenously added human recombinant TFF1 protein was sufficient to observe similar effects. Together, these findings suggest that the hypoxic conditions, which can be induced by gastric injury, promote TFF1 up-regulation, strengthened by an auto-induction mechanism, and that the trefoil peptide takes part in the epithelial-mesenchymal transition events eventually triggered to repair the damage.

## 1. Introduction

Trefoil factor family (TFF) comprises three secreted peptides synthesized by mucous-producing cells: the gastric peptide pS2/TFF1, the spasmolytic peptide (SP)/TFF2, and the intestinal trefoil factor (ITF)/TFF3. They share a conserved structural motif of 38 or 39 amino-acid residues with six cysteines that, through disulphide bonds, form a three-looped structure, the so-called ‘trefoil’ domain. TFFs are abundantly expressed in the gastrointestinal tract, where they protect mucous epithelia from different insults and contribute to epithelial continuity [1]; however, they display divergent effects depending on the patho-physiological state of the tissue that expresses them [2]. As a consequence, the role of TFF1 in cancer is still under debate because of its different expression and controversial behavior in various tumor contexts. Initially found in breast cancer cells [3], in this tumor type, TFF1 was indicated as an oncogene in some papers [4,5] and as a predictive factor of positive response to hormone therapy in some others [6,7]. Moreover, high levels of TFF1 were observed in different tumor tissues (colonic, pancreatic, and ovarian), in comparison with normal counterparts, correlated with the stimulation of cell survival, migration, invasiveness, and tumor dissemination [8,9,10].

Because TFF1-deficient mice develop antropyloric adenoma and 30% progress to gastric carcinoma [11], it is considered a specific gastric tumor-suppressor, but even in this case, there is controversial evidence. Several studies report a down-regulation of its expression because of deletions, mutations, or methylation of its promoter [12,13,14], and loss or reduced TFF1 expression is mostly associated with the intestinal and poorly differentiated histological sub-types [15,16]. In contrast, it is highly expressed in mucosa adjacent to gastric carcinoma compared with normal mucosa, in gastric mucosa with atypical hyperplasia and in diffuse-type and gastric carcinoma with nodal metastases [17,18,19].

In physiologic conditions, TFF1 is essential in maintaining the normal commitment program of epithelial progenitors in the oxyntic mucosa [20] and in the differentiation processes of the respiratory tract [21]. The trefoil protein protects gastric tissue from experimentally-induced damage, acting as a motogen to facilitate cell migration into the lesion in a process known as restitution [1]. Gastric mucosa is continuously exposed to injurious agents that can lead to damage of microvascular endothelium, impairment of oxygen delivery, and transport of nutrients. All these events result in mucosal erosion or ulceration, and tissue repair depends on the ability of epithelial cells to migrate and proliferate [22]. Wound healing and tissue regeneration are also associated with epithelial-mesenchymal transition events that are stimulated in order to reconstruct tissues following trauma and inflammatory injury [23].

As a protective factor, TFF1 was shown to be overexpressed in the mucosal ulceration of the digestive tract [24,25] and in the acute phase of *Helicobacter* infection [26]. Its expression is controlled at different levels by genetic and epigenetic mechanisms and depends from the methylation status of its promoter [27] and the presence of several transcription factors, including EGF, GATA6, AP-1, HNF3, and the copper-sensing transcription factor SP1 [28,29,30,31]. Hernández and coworkers [32] showed that under hypoxic conditions, the hypoxia inducible factor 1 (HIF-1) mediates the induction of the expression of TFF genes in gastric epithelial cells. Additionally, some studies described auto- and cross-induction mechanisms for TFF2 and TFF3 [33,34].

Hypoxia inducible factors, activated by reduced oxygen levels in a tumor microenvironment, trigger a set of adaptive responses commonly associated with tumor malignancy, including angiogenesis, a shift in metabolism, proliferation, invasion, and metastasis. In particular, HIF-1α is directly responsible for the epithelial to mesenchymal transition (EMT)-like changes of hypoxia-induced gastric cancer stem cells, which may result in the recurrence and metastasis of gastric cancer [35].

The purpose of our study was to explore the role of TFF1 in EMT and hypoxic conditions, processes inherently linked to inflammation, and tumor progression. Here, we describe a TFF1 auto-induction mechanism identifying a TFF1 responsive element in its promoter, suggesting its ability to work synergistically with HIF1-α under hypoxic conditions.

## 2. Results

### 2.1. TFF1 Overexpression Promotes Invasion and “EMT-Like” Molecular Changes

**Figure 1:**
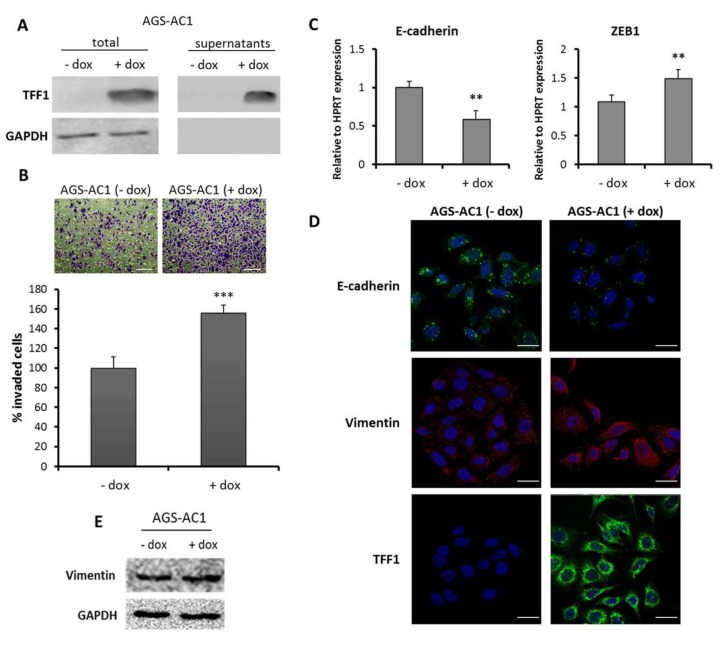
Trefoil factor 1 (TFF1) promotes invasion and epithelial to mesenchymal transition (EMT) changes in cellular models. (**A**) Protein level of TFF1 detected by western blotting. Protein normalization was performed on GAPDH levels; (**B**) Trans-well invasion assay of AGS-AC1 (TFF1 inducible hyperexpressing clone). Upper panel, bottom surface of filters stained with crystal violet. Magnification 10×. Bar = 100 μm. Lower panel, quantification of cell invasion. Statistically significant differences at *p <* 0.001 from the controls are indicated (***); (**C**) qRT-PCR for E-cadherin and ZEB1 mRNA expression in AGS-AC1 cells normalized on HPRT mRNA levels. Statistically significant differences at *p <* 0.01 from the non-induced cells are indicated (**); (**D**) Immunofluorescence analysis of TFF1 and vimentin on AGS-AC1 cells +/− doxycycline (induced or not induced to hyperexpress TFF1). Immunofluorescence images refer to 48 h after induction. Nuclei were stained with DAPI. Magnification 63×. Bar = 10 μm; (**E**) western blot analysis of vimentin expression in AGS-AC1 cells +/− doxycycline. Protein bands were normalized on GAPDH levels.

In order to analyze the effect of TFF1 restoration in a model system that does not express it, we used a TFF1 inducible hyper-expressing clone (AGS-AC1) derived from the gastric adenocarcinoma cell line AGS (Figure 1A). Numerous studies reported the ability of TFFs to stimulate migration and invasion of several cell lines. In our previous work, we demonstrated that TFF1 expression increases the migration of AGS-AC1 cells [36]. Here, we analyzed the effect of TFF1 on cell invasive ability. Trans-well invasion assay indicated that TFF1 hyper-expression significantly promoted the invasiveness of AGS-AC1 cells (Figure 1B). The invasion process results from various molecular and cellular mechanisms that overlap with EMT-inducing pathways [37]. During EMT, cells undergo molecular changes and gene expression shifts from an epithelial to a mesenchymal repertoire. To determine whether TFF1 hyper-expression was able to promote such a shift, we examined the expression of some EMT markers in AGS-AC1 cells after TFF1 induction. qRT-PCR showed an increased mRNA level of ZEB1, a central regulator of EMT [38], and reduced E-cadherin expression in AGS-AC1 TFF1 hyper-expressing cells, relative to the control cells (Figure 1C). Moreover, we also observed a cytoskeletal reorganization of the mesenchymal marker vimentin (Figure 1D), which weakly increases in AGS-AC1 cells after TFF1 induction (Figure 1E).

### 2.2. Exogenous TFF1 Protein Induces Invasion and EMT in AGS Cells

**Figure 2:**
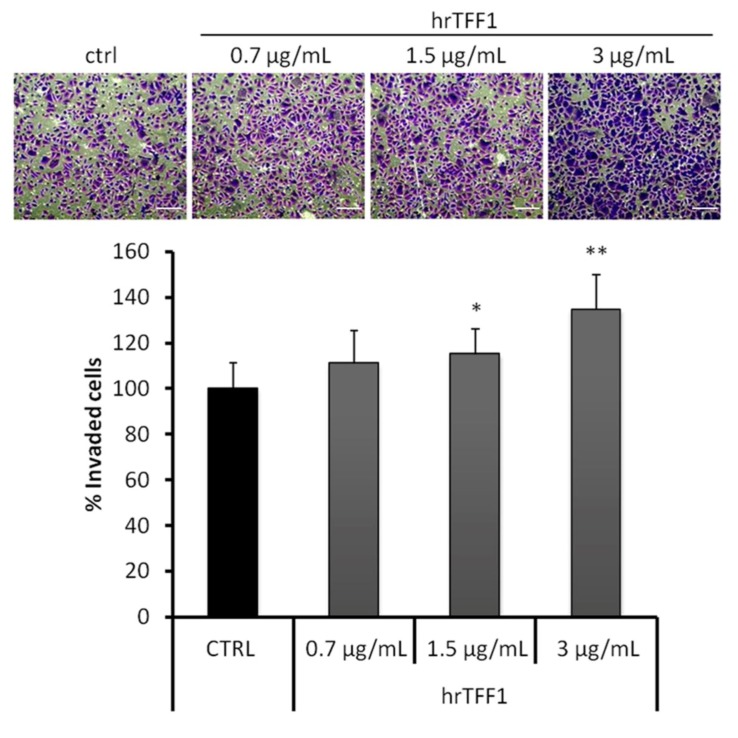
Recombinant TFF1 stimulates the invasive activity of AGS cells. Invasiveness rate of AGS treated or not with human recombinant dimeric TFF1 protein (hrTFF1) protein. Upper panel, bottom surface of filters stained with crystal violet. Magnification 10×. Bar = 100 μm. Lower panel, quantification of invasive cells. * *p <* 0.01 and ** *p <* 0.001 vs. untreated control.

TFF1 is a small secreted protein able to form homodimers, which are biologically more active than monomers [4,39]. To further understand the role of the protein in invasiveness and EMT switching, we examined the effect of exogenous human recombinant TFF1 dimeric protein on AGS cells, which do not express endogenous TFF1. Invasion assays were carried out in order to measure the cell-invading ability following the addition of human recombinant dimeric TFF1 protein (hrTFF1). We used different concentrations of hrTFF1 and observed that the invasive potential of AGS cells was significantly increased by TFF1 in a dose-dependent manner (Figure 2).

**Figure 3:**
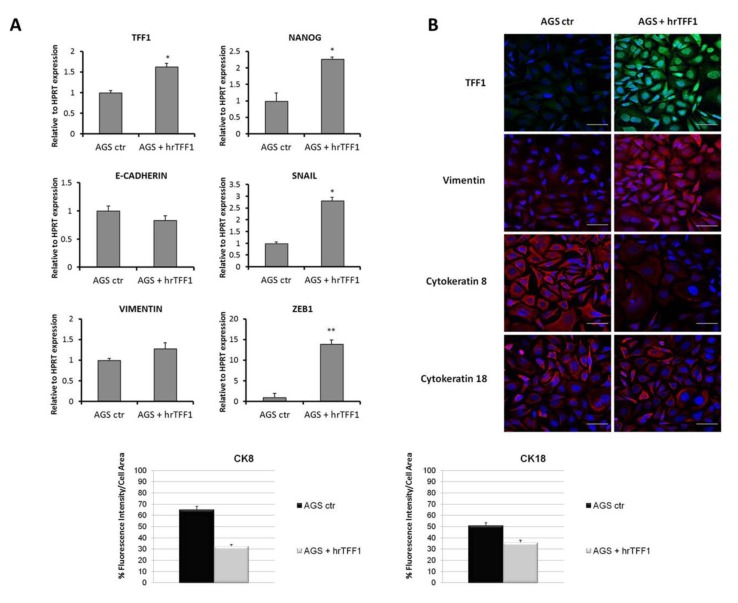
Effect of recombinant TFF1 on EMT markers expression in AGS cells. (**A**) mRNA expression of TFF1, Nanog, E-cadherin, Snail, vimentin, and ZEB1 detected and quantified by real-time PCR and correlated to the value of HPRT mRNA expression in AGS cells treated or not with hrTFF1 (4 μg/mL) for 72 h. * *p <* 0.05 and ** *p <* 0.01 vs. the untreated control; (**B**) Immunofluorescence analysis to detect TFF1, vimentin, and cytokeratin-8 and -18 on AGS cells treated or not with hrTFF1. Immunofluorescence images refer to 72 h after treatment. Nuclei were stained with DAPI. Magnification 63×. Bar = 10 μm; (**C**) Quantification of cytokeratin-8 and -18 as percent of fluorescence intensity per cell area from corresponding panels in Figure 3C. Statistical analyses for significance of results were performed using Student’s *t*-test, assuming a two-tailed distribution and unequal variance. * *p* < 0.05, ** *p* < 0.01.

Thereafter, we investigated the ability of exogenous TFF1 to induce EMT. AGS cells were treated for 72 h with hrTFF1 at a concentration of 3 μg/mL. We estimated that this TFF1 concentration was similar to that measured in AGS-AC1 supernatant (Figure S1). After treatments, recombinant protein was removed and cells were extensively washed. The elevated TFF1 signal showed in Figure 3B is essentially due to the internalization of the recombinant protein as already observed with the FITC-labeled one [31]. Treatment with recombinant TFF1 triggers EMT in AGS cells, causing a deregulation of several proteins and inducers involved in this process. In particular, qRT-PCR analysis showed significant up-regulation of Snail and ZEB1, well known repressors of E-cadherin expression, and Nanog, a stemness marker, while the epithelial marker E-cadherin showed a trend (even if non-statistically significant) to reduction and the mesenchymal one vimentin to induction (Figure 3A). Immunofluorescence analyses further confirmed an up-regulation and cytoskeletal reorganization of vimentin and a down-regulation of the epithelial markers cytokeratin-8 and -18 (Figure 3B,C).

### 2.3. TFF1 Potentiates Cell Invasiveness and EMT Changes during Hypoxia

**Figure 4:**
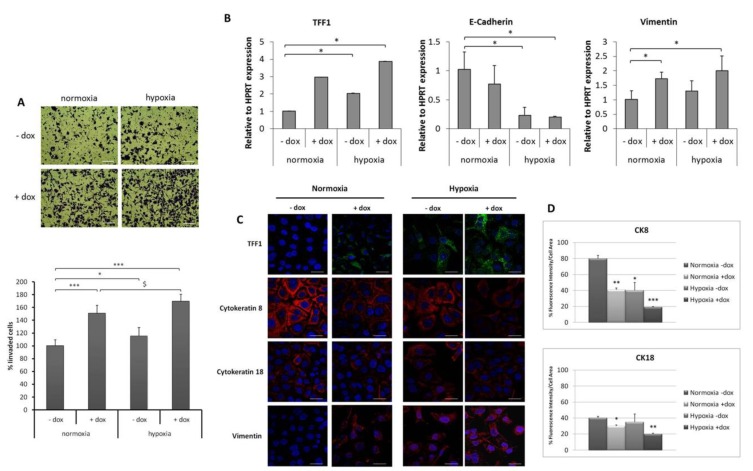
Effect of TFF1-enriched conditioned medium (CM) on the invasive ability of AGS cells and the expression of EMT markers. (**A**) Invasion through Matrigel of AGS cells treated with conditioned medium from AGS-AC1 cells (CM, −dox) or from TFF1 hyper-expressing AGS-AC1 cells (CM, +dox) and incubated for 24 h in normoxia or hypoxia. Upper panel, bottom surface of filters stained with crystal violet. Magnification 10×. Bar = 100 μm. Lower panel, quantification of invasive cells relative to normoxia. * *p <* 0.05 and *** *p <* 0.001 vs. normoxic CM, −dox controls, ^$^ *p <* 0.05 vs. AGS CM, +dox treated cells in normoxia; (**B**) qRT-PCR for TFF1, E-cadherin, and vimentin mRNA expression in AGS cells treated with CM, −dox or CM, +dox of AGS-AC1 cells under normoxia or hypoxia for 48 h. Data are normalized on mRNA levels of HPRT in the same experimental models. Statistically significant differences at *p <* 0.05 from normoxic CM, −dox controls are indicated (*); (**C**) Immunofluorescence images of TFF1, vimentin, and cytokeratin-8 and-18 on AGS cells treated with CM, −dox or CM, +dox of AGS-AC1 cells under normoxia or hypoxia for 48 h. Nuclei were stained with DAPI. Magnification 63×. Bar = 10 μm; (**D**) Quantification of cytokeratin-8 and -18 as percent of fluorescence intensity per cell area from corresponding panels in (**D**). Statistical analyses for significance of results were performed using Student’s *t*-test, assuming a two-tailed distribution and unequal variance. * *p* < 0.05, ** *p* < 0.01, *** *p* < 0.001.

The activation of hypoxia-inducible factor 1 (HIF-1α) has been demonstrated in a broad range of physiological responses to ischemic, hypoxic, and inflammatory conditions. As an example, gastric injury induced by non-steroidal anti-inflammatory drugs (NSAIDs) is associated with vascular damage that causes hypoxia [40]. Interestingly, the expression of TFFs has been demonstrated to be responsive to hypoxia through HIF-1 [32] and iNOS-derived NO associated with NSAID-induced gastric injury is implicated in mucosal restitution via the HIF-1-mediated induction of TFF genes [41]. To further explore TFF1’s role in EMT under hypoxic conditions, conditioned media (CM) were collected at 48 h of dox-induced and uninduced AGS-AC1 cells (in order to accumulate good levels of TFF1 in dox-induced ones) and transferred onto AGS cells for 24 or 48 h under normoxia or hypoxia. Our data showed that the cell invasion ability of AGS cells was enhanced under hypoxia compared with normoxia conditions. CM from TFF1 hyper-expressing AGS-AC1 cells (CM, +dox) significantly increased invasiveness of AGS during normoxia, and this effect was even enhanced upon hypoxic stress (Figure 4A). qRT-PCR analyses showed that CM from TFF1 hyper-expressing AGS-AC1 cells (CM, +dox) stimulated TFF1 expression in AGS cells both in normoxia and in hypoxia conditions compared with control cells (CM, −dox). At the same time, we observed a reduced expression of E-cadherin and an enhanced expression of vimentin that was more pronounced in TFF1-enriched hypoxic conditions (Figure 4B). Additionally, immunofluorescence experiments showed hypoxia down-regulation of the epithelial markers cytokeratin 8 and 18 and up-regulation of the mesenchymal one vimentin, and, in AGS treated with TFF1-enriched medium, we observed a potentiated effect (Figure 4C,D).

### 2.4. TFF1 Strengthens Its Own Induction upon Hypoxia Treatment

**Figure 5:**
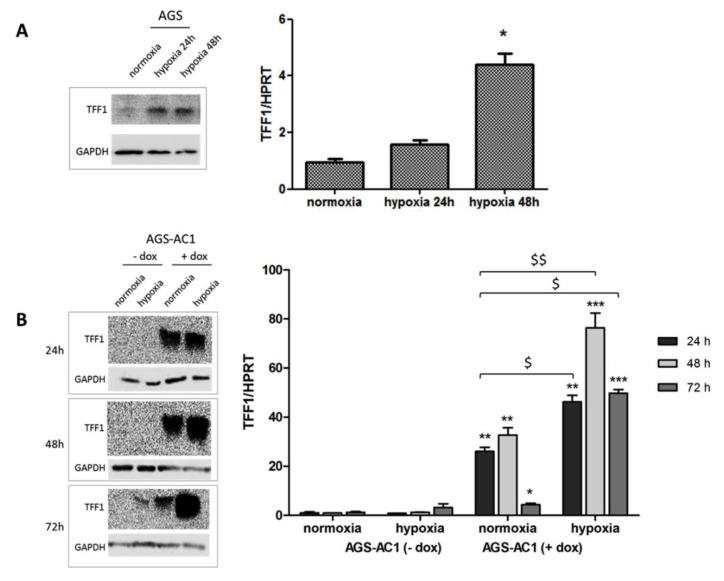
Hypoxia-induced TFF1 expression in cellular models. Expression of TFF1 in AGS cells after 24 and 48 h of hypoxia (**A**) and in AGS-AC1 cells after 24–48–72 h of hypoxia (**B**). Left panel, protein level of TFF1 detected by western blotting and normalized with corresponding GAPDH. Right panel, mRNA level of TFF1 by real-time PCR, measured on mRNA levels of HPRT in the same experimental models. * *p <* 0.05, ** *p <* 0.01 and *** *p <* 0.001 vs. AGS/AGS-AC1 cells in normoxia, ^$^ *p <* 0.05 and ^$$^ *p <* 0.01 vs. AGS-AC1 induced cells in normoxia.

Because TFF1 expression showed to be affected by hypoxic conditions in our model, we set up hypoxia experiments on AGS and AGS-AC1 cells, in order to measure TFF1 induction at different times from the hypoxic stimulus in cells expressing or not basal levels of the protein. Both TFF1 mRNA and protein expression levels increased significantly in response to hypoxia compared with the control cells maintained in normoxia. AGS cells, which typically express very low endogenous TFF1 levels, significantly induce TFF1 expression after 24 and 48 h of hypoxic stimulus (Figure 5A) concomitantly with the hypoxia inducible factor HIF-1α (Figure S2). In AGS-AC1 cells, qRT-PCR showed particularly elevated TFF1 mRNA levels at 48h and 72 h of hypoxia in TFF1 hyper-expressing cells (Figure 5B) if compared with normoxic conditions. The reduced TFF1 mRNA level at 72 h with respect to 48 h from dox induction is due to the exhausted effect of doxycycline also observed at the protein level (Figure S3). The fold induction of TFF1 hyper-expressing AGS-AC1 following hypoxia (up to 75-fold at 48 h of hypoxia) seems not to be simply the sum of the dox-induced mRNA (AGS-AC1 + dox in normoxic conditions, 30-fold) and hypoxia-induced endogenous mRNA (AGS-AC1 − dox in hypoxic conditions, two-fold), as it should be. This evidence suggested to us that TFF1 expression was induced not only by HIF-1α activation but also by TFF1 itself.

### 2.5. TFF1 Shows an Auto-Induction Mechanism

**Figure 6:**
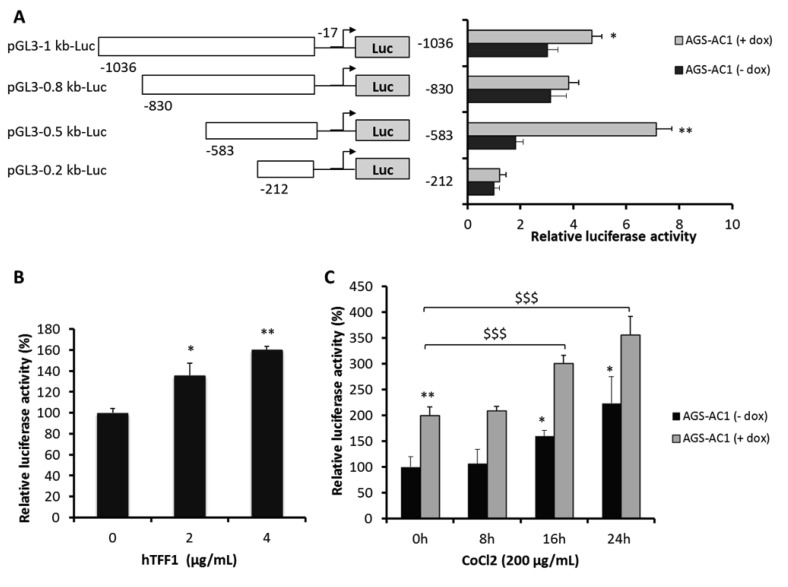
Regulation of the TFF1 promoter by TFF1 itself and hypoxia. (**A**) Schematic representation of luciferase reporter constructs containing fragments of varying lengths from −1036 to −212 bp of the TFF1 promoter upstream of a Luciferase reporter gene. Luciferase reporter assays in AGS-AC1 cells. TFF1 induction of cells was performed with doxycycline (1 μg/mL). * *p* < 0.05, ** *p* < 0.01 compared with non-induced cells expressing pGL3(−212)Luc; (**B**) Luciferase reporter assay with pGL3(583)Luc in AGS cells. At 48 h post-transfection, cells were stimulated with different concentrations of hrTFF1 (0–4 μg/mL) for 24 h. * *p <* 0.05, ** *p <* 0.01 vs. control cells (0 μg/mL); (**C**) Luciferase reporter assays with pGL3(−583)Luc and CoCl_2_ (200 μM) exposure for 8, 16, 24 h in AGS-AC1 cells. TFF1 induction of cells was performed with doxycycline (1 μg/mL). * *p* ≤ 0.05, ** *p* ≤ 0.01 vs. non-induced cells, ^$$$^ *p* ≤ 0.001 vs. induced cells. All data are representative of four different experiments reported as mean ± SD.

To get further insights on a possible auto-activation mechanism, we performed a luciferase reporter assay on AGS-AC1 cells. We transfected cells with a series of luciferase reporter-TFF1 promoter constructs, containing fragments corresponding to TFF1 promoter regions from −1036 bp, −830 bp, −583 bp, and −212 bp, upstream of a luciferase reporter gene. AGS-AC1 TFF1 hyper-expressing dox-induced cells showed luciferase activities higher than non-induced ones, indicating that TFF1 is able to auto-activate its own promoter (Figure 6A). In particular, the pGL3(−583)Luc construct gave the strongest luciferase up-regulation in AGS-AC1 induced cells, suggesting that a TFF1 responsive element was present between −583 and −212 bp of its promoter. Moreover, we could also hypothesize that an element able to suppress this activity is located between −830 and −583 bp. To confirm the effect of TFF1 on this 583 bp region of its own promoter, AGS cells were transiently transfected with the pGL3(−583)Luc construct and treated with different concentrations of hrTFF1. The promoter activity was significantly enhanced after hrTFF1 treatment in a dose-dependent manner (Figure 6B). As reported by Hernández and coworkers [32], TFF1 shows two hypoxia-responsive elements (HRE) specific for HIF-1α binding located at −552/−562 and −283/−293 bp from the transcription start site of its promoter. Our pGL3(−583)Luc construct contains both HIF-1α binding sites, so we performed luciferase assays on AGS-AC1 cells with the pGL3(−583)Luc construct mimicking hypoxia status with CoCl_2_ treatment. As expected, TFF1 overexpression significantly increased luciferase activity in AGS-AC1 cells (Figure 6C, zero time of incubation). Moreover, CoCl_2_ treatment increased reporter gene activity in TFF1 hyper-expressing cells (AGS-AC1 + dox) at higher levels compared to cells not expressing TFF1 (AGS-AC1 − dox) (Figure 6C).

### 2.6. TFF1 Regulates the Methylation Status of Its DNA

**Figure 7:**
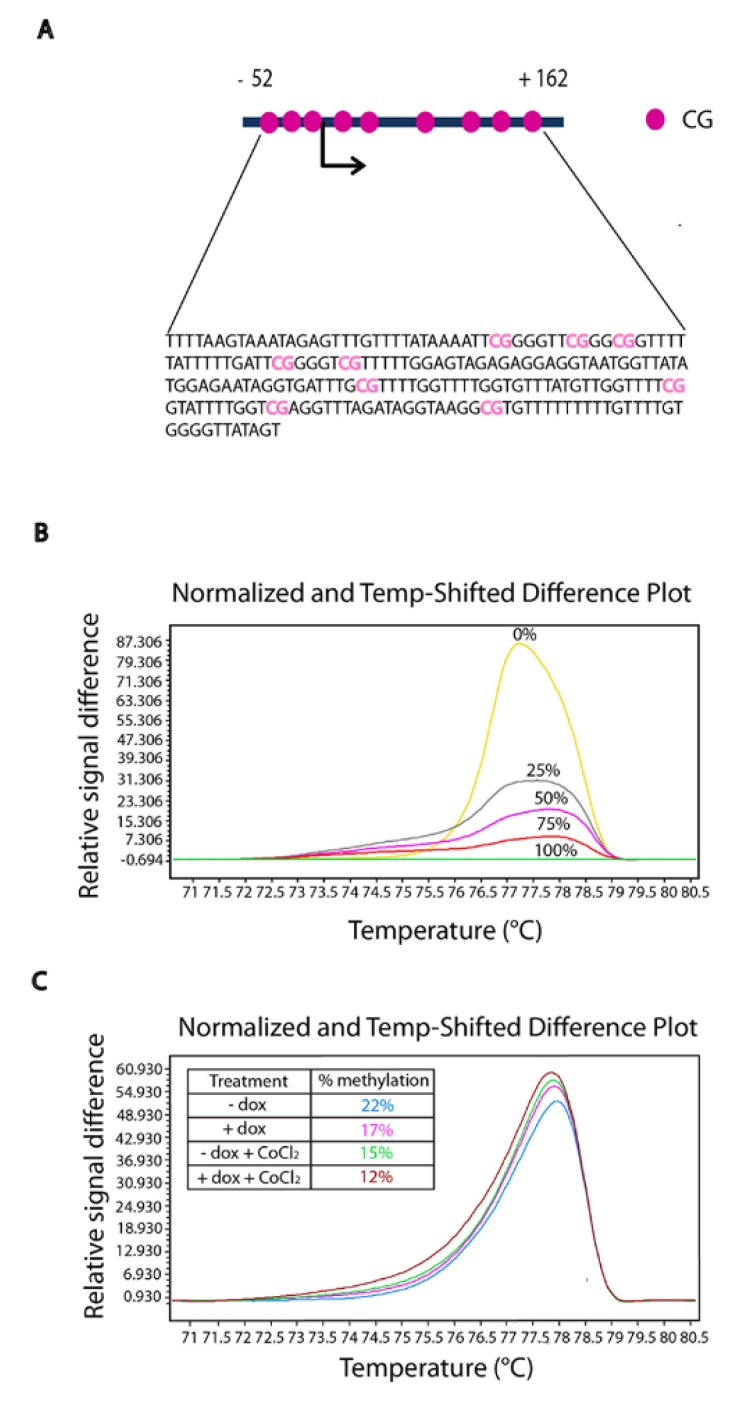
High-resolution melting (HRM) analysis of TFF1 promoter methylation. Difference plots of the TFF1 promoter DNA methylation pattern in AGS-AC1 cells after 72 h of induction with doxycycline (1 μg/mL) and/or with CoCl_2_ 200 μM. (**A**) TFF1 promoter region analyzed containing 9 CpG; (**B**) Normalized difference plot of control DNA samples with known percentage of methylation; (**C**) Normalized difference plot of AGS-AC1 DNA samples after treatments.

DNA methylation is an epigenetic mechanism that plays an important role in regulating gene expression, and TFF1 expression is strongly influenced by the methylation status of its promoter [12,42,43]; moreover, oxygen stress generates abnormal DNA methylation patterns [44]. Bisulfite modification was performed to explore the DNA methylation status of TFF1 in AGS-AC1 clone and to investigate its changes after TFF1 induction and in hypoxic condition. A high-resolution melting (HRM) analysis was performed on the TFF1 promoter in AGS-AC1 after 72 h from doxycyline induction and/or treatment with hypoxia-inducer CoCl_2_. Figure 7 shows that TFF1 hyper-expression (AGS-AC1 + dox) led to a lower level of methylation if compared with the control (AGS-AC1 − dox). Indeed, CoCl_2_ treatment also reduced the DNA methylation of the TFF1 promoter, and cells hyper-expressing TFF1 and maintained under hypoxia conditions showed a sum of the effects. These results suggested that TFF1 can auto-activate, at least partly, its own expression, regulating the density of methylated CpGs and that hypoxia, through HIF-1α activation, creates a loop with more TFF1 induction and subsequent lesser DNA methylation.

## 3. Discussion

TFF1 is a secretory peptide mainly expressed in the gastric mucosa, where it represents a protective player against mucosal damage. Mechanisms similar to epithelial–mesenchymal transition occur as a physiological response to injury: during wound healing, cells at the border of the wound recapitulate part of the EMT process and acquire an intermediate phenotype [45]. Because TFF1 was reported to be overexpressed in migratory cells at the edge of the wound [46], we hypothesize that it could be involved in an EMT-like mechanism of repair.

In this study, we investigated the impact of TFF1 on EMT and cell invasiveness, and we showed that TFF1 promotes the invasion of AGS cells and of an inducible TFF1 hyper-expressing cell clone, AGS-AC1, both in an autocrine and paracrine manner. Previously, we reported that the ectopic expression of TFF1 stimulates cellular migration [36]. Increased cellular migration and invasion abilities are characteristics of EMT. As expected, TFF1 is implicated in the occurrence of EMT in our cellular model, triggering the loss of some epithelial characteristics and the gaining of a mesenchymal-like phenotype. TFF1 hyper-expression or exogenous addition on gastric cancer cells led to the reduction of E-cadherin and of other epithelial markers, such as cytokeratins-8 and -18, and to the increase of mesenchymal markers, such as vimentin, and upregulation of significant transcription factors of EMT, such as Snail, ZEB1 and Nanog.

Several studies have previously examined the mechanisms regulating TFF1 expression. In particular, it has been demonstrated that TFFs genes expression is up-regulated in a HIF-1α-dependent manner during hypoxia [32]. Hypoxia is correlated with gastric injury, and it has been reported that damaging chemicals may induce severe vascular injury resulting in blood flow stasis, hypoxia, and necrosis of surrounding epithelial and mesenchymal cells [40,47]. Here, we report that a significant increase of TFF1 expression is associated with a hypoxia-related mesenchymal process. TFF1 is induced under hypoxic conditions as a target of HIF-1α and consequently promotes hypoxia-related cell invasion and EMT.

In addition, we focused on the mechanisms by which TFF1 can regulate these processes. Our experiments revealed the existence of an auto-regulatory mechanism that allowed TFF1 to control and sustain its own expression. The self-regulating mechanism seems to be a general property of the trefoil factor family. In gastric cell lines, the trefoil factors respond to auto- and cross-induction through cis-acting regulatory regions. A TFF2/SP response element has been identified within the 823 bp upstream of TFF2 transcriptional start site [33]. Moreover, recently, Sun and coworkers have identified the −1450 to −1400 bp fragment of the TFF3 promoter as the functional region for its self-induction [34]. We observed the enforced expression of TFF1-induced endogenous TFF1 mRNA expression. Luciferase reporter assays revealed that the TFF1 protein activates its own promoter activity in a dose-dependent manner, creating a self-induction loop. Additionally, we identified an auto-stimulatory element within the 0.6 kb (−583 to −17 bp) 5′-flanking region of the TFF1 promoter and demonstrated that it is responsive to the presence of the protein and able to positively regulate the expression of TFF1 also during hypoxia and synergistically with HIF1-α induction. We speculated that this auto-induction mechanism of TFF1 might be relevant to its action.

Furthermore, we investigated the epigenetic regulation of the trefoil protein in our cellular model, because DNA methylation plays a significant role in the control of its expression [48]. A recent study suggested that demethylation of a HIF-1α binding site in the HIF-1α promoter implements its auto-regulation [49]. To investigate if the same mechanism occurs for TFF1, we analyzed TFF1 promoter methylation by HRM. As expected, we observed that the methylation of TFF1 was reduced following hypoxia stimulus and/or self-regulatory mechanism. Decreased methylation correlated with its increased expression, suggesting that TFF1 expression is the result of closely linked mechanisms of regulation.

In conclusion, we showed that TFF1 triggers EMT-like changes in our cellular models and potentiates its effects under hypoxia through an auto-induction mechanism of regulation working together with HIF1α. We suggest that these molecular events can also aid to explain the double role of this protein in cancer initiation and progression: TFF1 loss in normal gastric tissue is well described to reduce mucosal protection against inflammatory processes predisposing to neoplasia, but TFF1 presence in a tumoral context could induce cancer progression.

Further experiments need to be performed to explore the involvement of TFF1 in EMT-like changes induced during the complex mechanism of repair in gastric tissues.

## 4. Materials and Methods

### 4.1. Cell Cultures

The human gastric cancer cell line AGS was purchased from the American Type Culture Collection (CRL-1739; Rockville, MD, USA). AGS cells were cultured in HAM’S F12 medium (Euroclone, Via Figino, Italy) supplemented with l-Glutamine 2 mM, 10% heat-inactivated fetal bovine serum (Euroclone), 100 U/mL of penicillin, and 100 µg/mL of streptomycin (Euroclone). The human clone hyper-expressing TFF1 under doxycycline induction (AGS-AC1) was selected from the AGS cell line as previously described [36]. The AGS-AC1 clone was cultured in DMEM medium (Euroclone) supplemented with 10% (*v*/*v*) fetal bovine serum, 100 U/mL penicillin, 100 µg/mL of streptomycin, and 600 µg/mL of neomycin (Euroclone). TFF1 expression was induced with 1 µg/mL of doxycycline. All cell lines were maintained at 37 °C in a 5% CO_2_ atmosphere.

### 4.2. Conditioned Media

Conditioned media (CM) were collected from AGS-AC1 cells after two days of induction or not with 1 µg/mL of doxycycline. Briefly, the cells were seeded at a density of 5 × 104 cells in DMEM complete medium into a 10-cm cell culture dish and grown to ~80% confluence. The cells were washed twice with phosphate-buffered saline (PBS), incubated in medium without FBS (for invasion assays) or with 2% FBS (for luciferase assays) without neomycin, and induced or not with doxycycline for 48 h. Then, CM were collected and centrifuged at 2000× *g* for 5 min to remove cell components. The presence of TFF1 in the conditioned media was assessed by western blot analysis.

### 4.3. Hypoxic Culture Conditions

Hypoxic conditions were obtained in two different ways: by incubating the cells in a modular incubator chamber (Billups-Rothenberg Inc., San Diego, CA, USA) flushed with a gas mixture containing 5% CO_2_ and 95% N_2_ at 37 °C or by treating the cells with the chemical inducer of hypoxia, CoCl_2_, 200 µM (Sigma Aldrich, Saint Louis, MO, USA).

### 4.4. Western Blot Analysis

Total intracellular proteins were extracted by freeze/thawing cells in lysis buffer (Tris HCl 20 mM, pH 7.4; sucrose 250 mM; DTT 1 mM; protease inhibitors, EDTA 1 mM). The protein content was estimated according to Bradford protein assay (BIO-RAD, Hercules, CA, USA). A total of 20 µg of proteins of each lysate was loaded on to a 10% SDS–PAGE and then transferred onto nitrocellulose membrane (Immobilon-NC, Millipore, Burlington, MA, USA). The membranes were blocked with 5% non-fat dry milk (BioRad) in -Tween (NaCl 150 mM; KCl 3 mM; Tris-HCl 25 mM pH 8, 0.1% Tween 20) and then incubated overnight at 4 °C with the primary antibodies. Antibodies against TFF1 (rabbit polyclonal; 1:500; GenScripts Corp, Piscataway, NJ, USA), vimentin (mouse monoclonal, clone E-5; 1:5000; Santa Cruz Biotechnologies, Dallas, TX, USA), HIF1-α (rabbit polyclonal; clone A300-286A; 1:5000; Bethyl Laboratories, Montgomery, TX, USA), GAPDH (mouse monoclonal; 1:1000; Santa Cruz Biotechnologies) were used. The proteins were visualized using enhanced chemioluminescence reagents (GE Healthcare Life Sciences, Marlborough MA, USA) after incubation at room temperature with an appropriate secondary rabbit or mouse antibody (1:5000; Sigma-Aldrich). The blots were exposed to LAS 4000 (GE Healthcare Life Sciences, Pittsburgh, PA, USA) digital imaging system, and the relative band intensities were determined using ImageQuant software (GE Healthcare Life Sciences).

### 4.5. Confocal Microscopy

The cells grown on coverslips were fixed with 4% *p*-formaldehyde for 5 min at room temperature, permeabilized with 0.5% Triton X-100 for 5 min, and blocked for 30 min with goat serum (20% *v*/*v* PBS; Lonza, Basel, Switzerland). The coverslips were incubated with rabbit anti-TFF1 antibody (1:500; Life Span Biosciences, Seattle, WA, USA), mouse anti-vimentin (1:500; Santa Cruz Biotechnologies), or mouse anti-CK8 or anti-CK18 (1:1000; Santa Cruz Biotechnologies) overnight at 4 °C. After two washing steps with PBS, the coverslips were incubated with anti-rabbit and/or anti-mouse AlexaFluor (488 and/or 555; 1:1000; Molecular Probes, Eugene, OR, USA) for 2 h at room temperature and then mounted with Mowiol (Mowiol 4-88, Sigma-Aldrich). Hoechst 33,342 (Molecular Probes, Eugene, OR, USA) was used to detect nuclei. A Zeiss LSM 710 Laser Scanning Microscope (Carl Zeiss MicroImaging GmbH, Göttingen, Germany) was used for data acquisition. The images were processed using ImageJ software (NIH), Adobe Photoshop CS version 5.0, and the figures were assembled using Microsoft PowerPoint (Microsoft Corporation, Redmond, WA, USA). The quantifications were performed on multichannel images obtained from a 63× objective using ImageJ, marking either the cell perimeter or the nucleus as the region of interest and calculating the integrated densities per area from the appropriate channel. A minimum of 30 cells were analyzed for each data set. The obtained mean value was used to compare experimental groups.

### 4.6. Quantitative Real-Time Polymerase Chain Reaction (qRT-PCR)

mRNA levels were analyzed by qRT-PCR using the Light Cycler 480 II instrument (Roche, Basel, Switzerland). The total RNA was extracted from the cultured cells using TriPure Isolation Reagent (Roche). Next, 1 µg of total RNA was reverse transcribed into cDNA with M-MLV Reverse Transcriptase kit (Sigma). Then, 5 µL of 1:10 diluted cDNA were used in a 20 µL reaction using StoS Quantitative Master Mix 2X SYBR Green (GeneSpin, Via Friuli, Italy). The quantitative measurements were analyzed using the comparative 2-∆∆*C*_t_ method. The primer sequences are reported in Table S1.

### 4.7. Trans-Well Invasion Assay

AGS and AGS-AC1 invasiveness was analyzed using Trans-well Cell Culture (12 mm diameter, 8.0-μm pore size; Corning Incorporated; Corning, NY, USA). The membranes of the upper chambers were coated with Matrigel (Becton Dickenson, Franklin Lakes, NJ, USA) and placed in a well containing media supplemented with 10% FBS. The cells were seeded at a number of 9 × 10^4^/insert into the upper chambers in serum-free medium. Treatments with recombinant TFF1 protein (hrTFF1, Raybiotech, Norcross, GA, USA) and conditioned media were carried out in the lower chamber. After 24 h of incubation at 37 °C in 5% CO_2_–95% air humidified atmosphere, the filters were fixed with 4% *p*-formaldehyde for 10 min and then with 100% methanol for 20 min. The cells on the lower surface of the filter were stained with a 0.5% crystal violet solution. The cells migrating to the lower surface were counted in twelve random fields using EVOS light microscope (10×) (Life technologies Corporation, Carlsbad, CA, USA).

### 4.8. Plasmid Constructs and Luciferase Assays

A 1 kb region (−1036 to −17 bp) upstream of TFF1 gene was amplified by PCR on genomic DNA extracted from breast cancer cells (MCF-7) and cloned into the pGL3 vector, obtaining pGL3(−1036)Luc. Sequential deletions by Bal31 exonuclease digestions were performed obtaining the subsequent plasmids: pGL3(−830)Luc (from −830 to −17 bp), pGL3(−583)Luc (from −583 to −17 bp), and pGL3(−212)Luc (from −212 to −17 bp).

AGS and AGS-AC1 cells were seeded in a 24-well plate at a number of 8 × 104/well and after 24 h transfected using Lipofectamine 2000 reagent (Invitrogen, Carlsbad, CA, USA) with 0.2 µg of plasmid constructs. A β-galactosidase control vector (Promega, Madison, WI, USA) (0.1 µg/well) was used for standardization. Then, 6 h after transfection, the medium was replaced, and the AGS-AC1 cells were induced with doxycycline. Next, 48 h after transfection, the AGS-AC1 cells were incubated in the presence or absence of 200 µM of CoCl_2_ (Sigma Aldrich) and AGS cells with 0–4 μg/mL of TFF1 recombinant protein (hrTFF1, Raybiotech, Norcross, GA, USA). Luciferase activity was measured using the Luciferase/β-Galactosidase Luciferase Assay Kit, Dual-Light (Applied Biosystems, Foster City, CA, USA), according to the manufacturer’s instructions. The experiment was performed in quadruplicate and the results are reported as the ratio between firefly luciferase and beta-galactosidase activity. The light emission was measured with EnSpire Multimode Plate Reader (PerkinElmer, Waltham, MA, USA).

### 4.9. High-Resolution Melting (HRM) Analysis

Genomic DNA was purified from cells with a NucleoSpin^®^ Tissue kit (Macherey Nagel, Düren, Germany) and quantized with NanoDrop 1000 (Thermo Scientific, Waltham, MA, USA). The bisulfite modification was carried out using an EZ DNA Methylation™ kit (Zymo Research, Irvine, CA, USA) on 500 ng of genomic DNA. A pair of CpG-free primers: (forward, 5′-TTTTAAGTAAATAGAGTTTGTTTTATAAAAT-3′; reverse, 5′-ACTATAACCCCACAAAACAAAAAAAAA-3′) was designed to amplify a 214 bp fragment containing 9 CpG (from −52 to +162 bp) around the transcription start site of the TFF1 promoter. The reactions were prepared in 96-well plates (LightCycler^®^ 480, Roche), using a LightCycler^®^ 480 High-Resolution Melting Master kit (Roche). The amplification and melting analyses were performed with the LightCycler^®^ 480 System. PCR comprises a step of 10 min at 95 °C, followed by 45 cycles of 10 s at 95 °C, 10 s at the annealing temperature, and 15 s at 72 °C. HRM profiles were obtained increasing temperatures from 65 to 95 °C (0.02 °C/s). The row melting data were analyzed with LightCycler^®^ 480 Gene Scanning Software, obtaining the difference melting curves. In order to measure the percentage of methylation in our samples, we generated a standard curve. CpG Genome™ Universal Methylated DNA (Millipore) was used as 100%, while DNA from MCF-7 was used as 0% (for the TFF1 promoter methylation [50]). The two standards were mixed to obtain 0%, 25%, 50%, 75%, 90%, and 100% TFF1 promoter methylation. Data from normalized, temperature-shifted, difference curves were exported to Excel for further quantification. A linear regression analysis was used to generate a standard curve from the peak heights of known methylated samples and to determine the degree of methylation of each DNA sample [51].

### 4.10. Statistical Analysis

All results are the mean ± SD of at least three experiments. The optical density of the protein bands detected by western blotting was normalized against GAPDH or β-actin levels. Statistical comparisons between the groups were made using an unpaired, two-tailed *t*-test, comparing two variables. The differences were considered significant if *p <* 0.05 and *p <* 0.01.

## Supplementary Materials

Supplementary materials can be found at http://www.mdpi.com/1422-0067/19/7/2018/s1.
