# Supplementary material for: TFF1 Promotes EMT-Like Changes through an Auto-Induction Mechanism
Source: Int J Mol Sci. 2018 Jul 11;19(7):2018. doi: 10.3390/ijms19072018 (PMC6073196; doi:10.3390/ijms19072018)
Supplement: Supplementary file 1 [file ijms-19-02018-s001.pdf]

International Journal of
Molecular Sciences

MDPI

TFF1 Promotes EMT-Like Changes through an Auto-Induction Mechanism

Supplementary Materials

Table S1. Primers sequences used for real-time PCR analysis.

Gene	Forward (5′-3′)	Reverse (5′-3′)	T annealing °C
E-CADHERIN	TTCCCAACTCCTCTCCTG	AAACCTTGCCTTCTTTGTC	58
HPRT1	GACCAGTCAACAGGGACAT	CCTGACCAAGGAAAGCAAAG	60
NANOG	CAGTCTGGACACTGGCTGAA	CTCGCTGATTAGGCTCCAAC	60
SNAIL	TGCCCTCAAGATGCACATCCGA	GGGACAGGAGAAGGGCTTCTC	60
TFF1	GGACGTCGATGGTATTAGGATA	AGGAGGTAATGGCCACCATGGA	58
VIMENTIN	CTCCGGGAGAAATTGCAGGA	TTCAAGGTCAAGACGTGCCA	60
ZEB1	GCCAATAAGCAAACGATTCTG	TTTGGCTGGATCACTTTCAAG	60

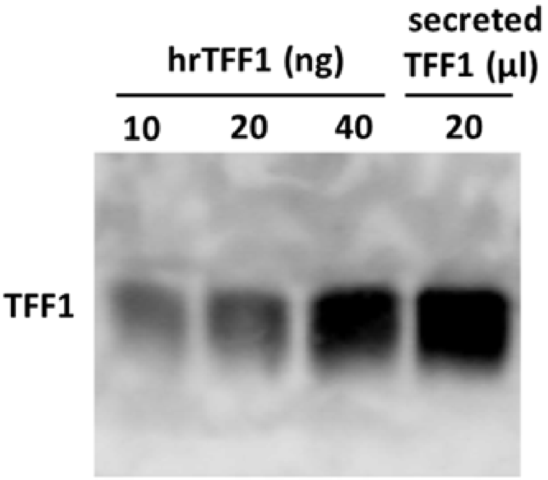

Figure S1. Western blot analysis of human recombinant TFF1 protein (10, 20 and 40 ng) and TFF1 secreted protein in AGS-AC1 supernatant after doxycycline induction (20 µl).

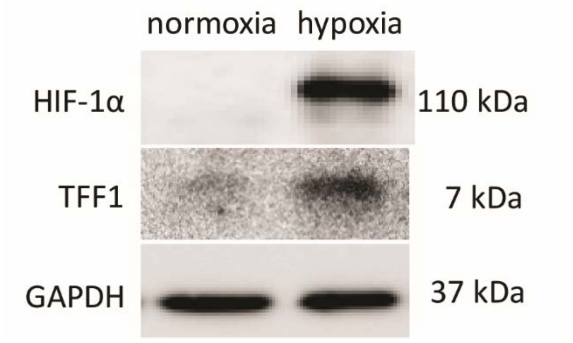

Figure S2. Western blot analysis of HIF-1α and TFF1 protein levels in AGS cell line under normoxic and hypoxic conditions.

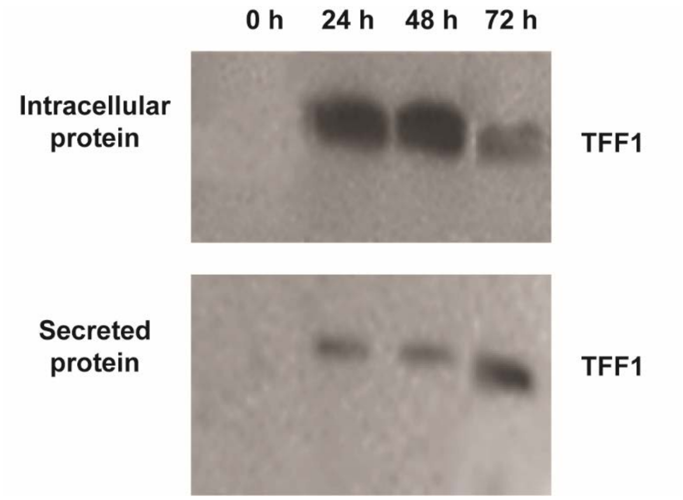

Figure S3. Time course analysis of TFF1 production in AGS AC1 cell line performed on intracellular and secreted protein at different times from induction with Doxycycline.
